# In silico molecular docking and in vitro antimicrobial efficacy of phytochemicals against multi-drug-resistant enteroaggregative *Escherichia coli* and non-typhoidal *Salmonella* spp.

**DOI:** 10.1186/s13099-021-00443-3

**Published:** 2021-07-17

**Authors:** Padikkamannil Abishad, Pollumahanti Niveditha, Varsha Unni, Jess Vergis, Nitin Vasantrao Kurkure, Sandeep Chaudhari, Deepak Bhiwa Rawool, Sukhadeo Baliram Barbuddhe

**Affiliations:** 1 Department of Veterinary Public Health, College of Veterinary and Animal Sciences, KVASU, 673 576 Pookode, Wayanad, India; 2 ICAR-National Research Centre on Meat, Chengicherla, Boduppal Post, 500 092 Hyderabad, India; 3 Nagpur Veterinary College, MAFSU, Seminary Hills, 440 006 Nagpur, India

**Keywords:** Phytochemical, Docking, Enteroaggregative *E. coli*, Non-typhoidal *Salmonella*

## Abstract

**Background:**

In the wake of emergence of antimicrobial resistance, bioactive phytochemical compounds are proving to be important therapeutic agents. The present study envisaged in silico molecular docking as well as in vitro antimicrobial efficacy screening of identified phytochemical ligands to the dispersin (aap) and outer membrane osmoporin (OmpC) domains of enteroaggregative *Escherichia coli* (EAEC) and non-typhoidal *Salmonella* spp. (NTS), respectively.

**Materials and methods:**

The evaluation of drug-likeness, molecular properties, and bioactivity of the identified phytocompounds (thymol, carvacrol, and cinnamaldehyde) was carried out using Swiss ADME, while Protox-II and StopTox servers were used to identify its toxicity. The in silico molecular docking of the phytochemical ligands with the protein motifs of dispersin (PDB ID: 2jvu) and outer membrane osmoporin (PDB ID: 3uu2) were carried out using AutoDock v.4.20. Further, the antimicrobial efficacy of these compounds against multi-drug resistant EAEC and NTS strains was determined by estimating the minimum inhibitory concentrations and minimum bactericidal concentrations. Subsequently, these phytochemicals were subjected to their safety (sheep and human erythrocytic haemolysis) as well as stability (cationic salts, and pH) assays.

**Results:**

All the three identified phytochemicals ligands were found to be zero violators of Lipinski’s rule of five and exhibited drug-likeness. The compounds tested were categorized as toxicity class-4 by Protox-II and were found to be non- cardiotoxic by StopTox. The docking studies employing 3D model of dispersin and ompC motifs with the identified phytochemical ligands exhibited good binding affinity. The identified phytochemical compounds were observed to be comparatively stable at different conditions (cationic salts, and pH); however, a concentration-dependent increase in the haemolytic assay was observed against sheep as well as human erythrocytes.

**Conclusions:**

In silico molecular docking studies provided useful insights to understand the interaction of phytochemical ligands with protein motifs of pathogen and should be used routinely before the wet screening of any phytochemicals for their antibacterial, stability, and safety aspects.

**Supplementary Information:**

The online version contains supplementary material available at 10.1186/s13099-021-00443-3.

## Background

Food-borne illnesses constitute a nagging public health issue that causes considerable impediments to the global health and economy, owing to the globalization and active food trade across countries [1]. Contamination of foods can lead to food-borne illnesses that can occur at any point of production, processing, distribution, and consumption. This emerging public health problem causes considerable obstruction to socio-economic development as well as contributes significantly to the global burden of disability, morbidity, and mortality. Global estimates correlated foodborne illnesses with nearly 600 million episodes, 4,20,000 mortality, and 33 million disability-adjusted life years [2].

Enteric bacterial pathogens namely, *Salmonella* spp. and *Escherichia coli* are important ‘priority’ listed foodborne pathogens [3]. Non-typhoidal *Salmonella* (NTS) serovars and diarrhoeagenic *E. coli* (DEC) pathotypes constitute the leading causes of gastrointestinal infections worldwide [4, 5]. Globally, the NTS serovars are responsible for approximately 153 million cases of gastroenteritis and 7000 deaths annually [6]. Besides, the DEC pathotypes especially, enteroaggregative *E. coli* (EAEC), are long been associated with foodborne outbreaks globally, thereby posing risk to global food safety and public health [5, 7]. The occurrence of NTS and EAEC has widely been reported from both developed as well as developing countries [4, 5, 8]. Antimicrobials have recently been employed on a larger scale as prophylactic as well as therapeutic agents to combat infections [9]. In recent times, the evolution and natural selection of bacteria along with the unprecedented use of antimicrobials have contributed to an alarming increase in antimicrobial resistance (AMR) [10]. Moreover, it has also been estimated that the mortality rate by way of AMR would increase to the tune of 10 million by 2050, which would further decrease the gross domestic product (GDP) by 3.50 %, resulting in an overall global economic loss of nearly USD 100 trillion [11]. Of late, multi-drug resistance among the NTS serotypes and EAEC has been reported from various sources [5, 12]. Hence, the focus has primarily been shifted towards alternative therapeutic strategies to counter the menace of AMR, apart from the routinely employed antibiotics [13].

Recently, the use of phytochemicals has emerged as one of the promising holistic alternative approaches with minimal side effects [14]. The bioactive phytochemicals and essential oils were found to have exerted significant antimicrobial activity against *Salmonella* spp. and *E. coli* [15]. The screening of molecules with potential bioactivity is quite costly and may consume time. However, computer-aided drug design (CADD) could save time as well as the cost of synthesis of molecules and would ultimately curtail the cost of research [16]. In silico molecular docking is one such CADD technique that would virtually predict the binding efficacy as well as the structure-based drug design [16]. Moreover, the molecular docking provides successful insights into the structure-activity relationships, mode of activity, and further analysis from protein-ligand interaction [17]. Such studies would culminate in the development of novel drug molecules at a faster pace against infectious pathogens. Additionally, the physicochemical properties of the molecule would provide vital information on the initial phase of drug development [16, 17].

The phytochemical compounds—monoterpenoids (thymol and carvacrol) and phenylpropanoid (cinnamaldehyde) are generally considered safe for human consumption and have been approved by Food and Drug Administration (FDA) for being used as food additives. Moreover, these phytocompounds have been extracted from various indigenous herbs located in different parts of India [18–21]. The objective of the present study was to study in silico absorption, distribution, metabolism, excretion, and toxicity (ADMET) analysis of the identified phytochemicals viz., thymol, carvacrol, and cinnamaldehyde, followed by in silico blind docking approach to check their ligand binding affinities to the outer membrane osmoporin protein (ompC) of *Salmonella* Enteritidis and/or *Salmonella* Typhimurium, and dispersin (aap) domain of EAEC. Later, the in silico approach was vetted by performing in vitro antimicrobial efficacy of these phytochemicals against the multi-drug resistant (MDR)-strains of NTS and EAEC. Also, in vitro safety and stability aspects of the identified phytochemicals were explored.

## Results

The EAEC (n = 3) and NTS (n = 3) strains used in this study were multi-drug resistant. The results of antibiotic susceptibility testing of the test strains are given as Additional file 1: Table S1.

### ADMET analysis

The ADME analysis (physicochemical properties, water solubility, lipophilicity, pharmacokinetics, drug likeness and medicinal chemistry) of the three phytochemicals tested (thymol, carvacrol and cinnamaldehyde) was carried out by Swiss ADME software (Table 1).

**Table 1:** In silic﻿o ADME analysis of phytochemicals tested

Sl no.	Descriptors	Thymol	Carvacrol	Cinnamaldehyde
*Physicochemical properties*				
1.	SMILE	Cc1ccc(c(c1)O)C(C)C	CC(c1ccc(c(c1)O)C)C	O=CC=Cc1ccccc1
2.	Formula	C10H14O	C10H14O	C9H8O
3.	Molecular weight (g/mol)	150.22	150.22	132.16
4.	Number of heavy atoms	11	11	10
5.	Number of aromatic heavy atoms	6	6	6
6.	Fraction Csp3	0.40	0.40	0.00
7.	Number of rotatable bonds	1	1	2
8.	Number of H-bond acceptors	1	1	1
9.	Number of H-bond donors	1	1	0
10.	Molar refractivity	48.01	48.01	41.54
11.	Topological polar surface area (Å²)	20.23	20.23	17.07
*Lipophilicity*				
12.	Log *P*_o/w_ (iLOGP)	2.32	2.24	1.65
13.	Log *P*_o/w_ (XLOGP3)	3.30	3.49	1.90
14.	Log *P*_o/w_ (WLOGP)	2.82	2.82	1.79
15.	Log *P*_o/w_ (MLOGP)	2.76	2.76	2.01
16.	Log *P*_o/w_ (SILICOS-IT)	2.79	2.79	2.48
17.	Consensus log *P*_o/w_	2.80	2.82	1.97
*Water solubility*				
18.	Log *S* (ESOL)	− 3.19	− 3.31	− 2.17
19.	Solubility	9.74e−02 mg/ml; 6.49e−04 mol/l	7.40e−02 mg/ml; 4.92e-04 mol/l	8.97e−01 mg/ml; 6.79e−03 mol/l
20.	Class	Soluble	Soluble	Soluble
21.	Log *S* (Ali)	− 3.40	− 3.60	− 1.88
22.	Solubility	5.97e−02 mg/ml; 3.98e−04 mol/l	3.79e−02 mg/ml; 2.53e−04 mol/l	1.74e + 00 mg/ml; 1.31e−02 mol/l
23.	Class	Soluble	Soluble	Very soluble
24.	Log *S* (SILICOS-IT)	− 3.01	− 3.01	− 2.40
25.	Solubility	1.46e-01 mg/ml; 9.71e−04 mol/l	1.46e−01 mg/ml; 9.71e−04 mol/l	5.26e−01 mg/ml; 3.98e−03 mol/l
26.	Class	Soluble	Soluble	Soluble
*Pharmacokinetics*				
27.	GI absorption	High	High	High
28.	BBB permeant	Yes	Yes	Yes
29.	P-gp substrate	No	No	No
30.	CYP1A2 inhibitor	Yes	Yes	No
31.	CYP2C19 inhibitor	No	No	No
32.	CYP2C9 inhibitor	No	No	No
33.	CYP2D6 inhibitor	No	No	No
34.	CYP3A4 inhibitor	No	No	No
35.	Log *K*_p_ (skin permeation) (cm/s)	− 4.87	− 4.74	− 5.76
*Drug-likeness*				
36.	Lipinski	Yes; 0 violation	Yes; 0 violation	Yes; 0 violation
37.	Ghose	No; 1 violation: MW < 160	No; 1 violation: MW < 160	No; 2 violations: MW < 160, #atoms < 20
38.	Veber	Yes	Yes	Yes
39.	Egan	Yes	Yes	Yes
40.	Muegge	No; 2 violations: MW < 200, Heteroatoms < 2	No; 2 violations: MW < 200, Heteroatoms < 2	No; 2 violations: MW < 200, Heteroatoms < 2
41.	Bioavailability score	0.55	0.55	0.55
*Medicinal chemistry*				
42.	PAINS	0 Alert	0 Alert	0 Alert
43.	Brenk	0 Alert	0 Alert	2 Alerts: aldehyde, michael_acceptor_1
44.	Lead likeness	No; 1 violation: MW < 250	No; 1 violation: MW < 250	No; 1 violation: MW < 250
45.	Synthetic accessibility	1.00	1.00	1.65

All the phytochemicals tested revealed drug likeness with no violation to Lipinski’s rule of five. Also, all the phytochemical compounds exhibited a bioavailability score of 0.55, indicating their drug- like properties. Besides, all the phytochemicals under study were found to be absorbed in the gastrointestinal tract and could cross the blood brain barrier. The topological polar surface area ranged from 17.07 to 20.23 Å², while the consensus log Po/w (indicator of lipophilicity) was observed to range from 1.97 to 2.82. Besides, the compounds tested exhibited no permeability glycoprotein substrate (P-gp). Additionally, all the phytochemicals tested interacted only with CYP1A2 isoenzyme of cytochrome P family, conferring their effectiveness with minimal toxicity (Table 1).

The bioavailability radar plots of the tested phytochemicals (Additional file 1: Fig. S1) indicated that the phytochemicals were fairly inside the pink area, indicating their drug-likelihood with a better bioavailability profile. Additionally, the boiled egg graph of thymol, carvacrol and cinnamaldehyde appeared within the yellow region (yolk) with a red point, predicting their brain penetrability acting as a non-substrate of P-gp (Additional file 1: Fig. S2).

Further, the in-silico toxicity properties of the phytochemicals were evaluated by Protox- II and StopTox softwares (Table 2). The predicted LD_50_ (mg/kg) for all the phytochemicals tested ranged from 640 to 1850; hence, were categorized as toxicity class-4 by Protox-II. Further, all the tested phytocompounds were found to be non-cardiotoxic by StopTox software, based on hERG liability prediction, with the confidence levels ranges between 50 and 60 % (Table 2).

**Table 2:** In silico toxicity analysis of phytochemicals tested

Sl no.	Phytochemicals	Oral toxicity of phytochemicals (*PROTOX II*)		hERG liability prediction/ confidence (*StopTox*)
		Predicted LD50 (mg/kg)	Predicted toxicity class	
1.	Thymol	640	4	Non-cardiotoxic (60)
2.	Carvacrol	810	4	Non-cardiotoxic (50)
3.	Cinnamaldehyde	1850	4	Non-cardiotoxic (60)

### Molecular docking using Autodock v.4.20

**Fig. 1:**
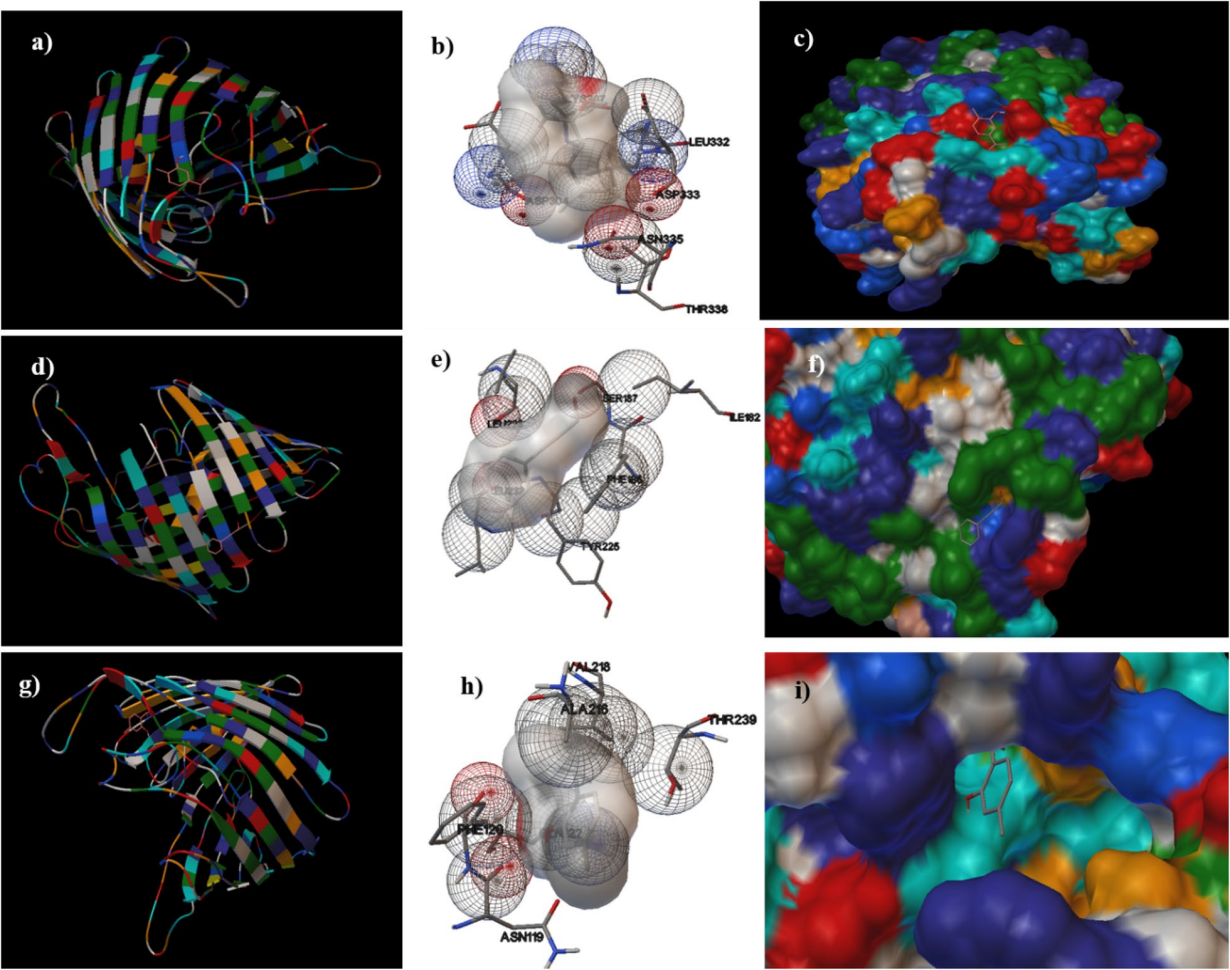
2-D and 3-D interactions of molecular docking of tested phytochemicals inside ompC. The horizontal rows denote interactions with carvacrol (**a**–**c**), cinnamaldehyde (**d**–**f**) and thymol (**g**–**i**), respectively; **a**, **d**, **g** denotes secondary structures of protein–ligand complexes; **b**, **e**, **h** protein–ligand interaction, whereas **c**, **f**, **i** denotes 3-D conformation of complexes

**Fig. 2:**
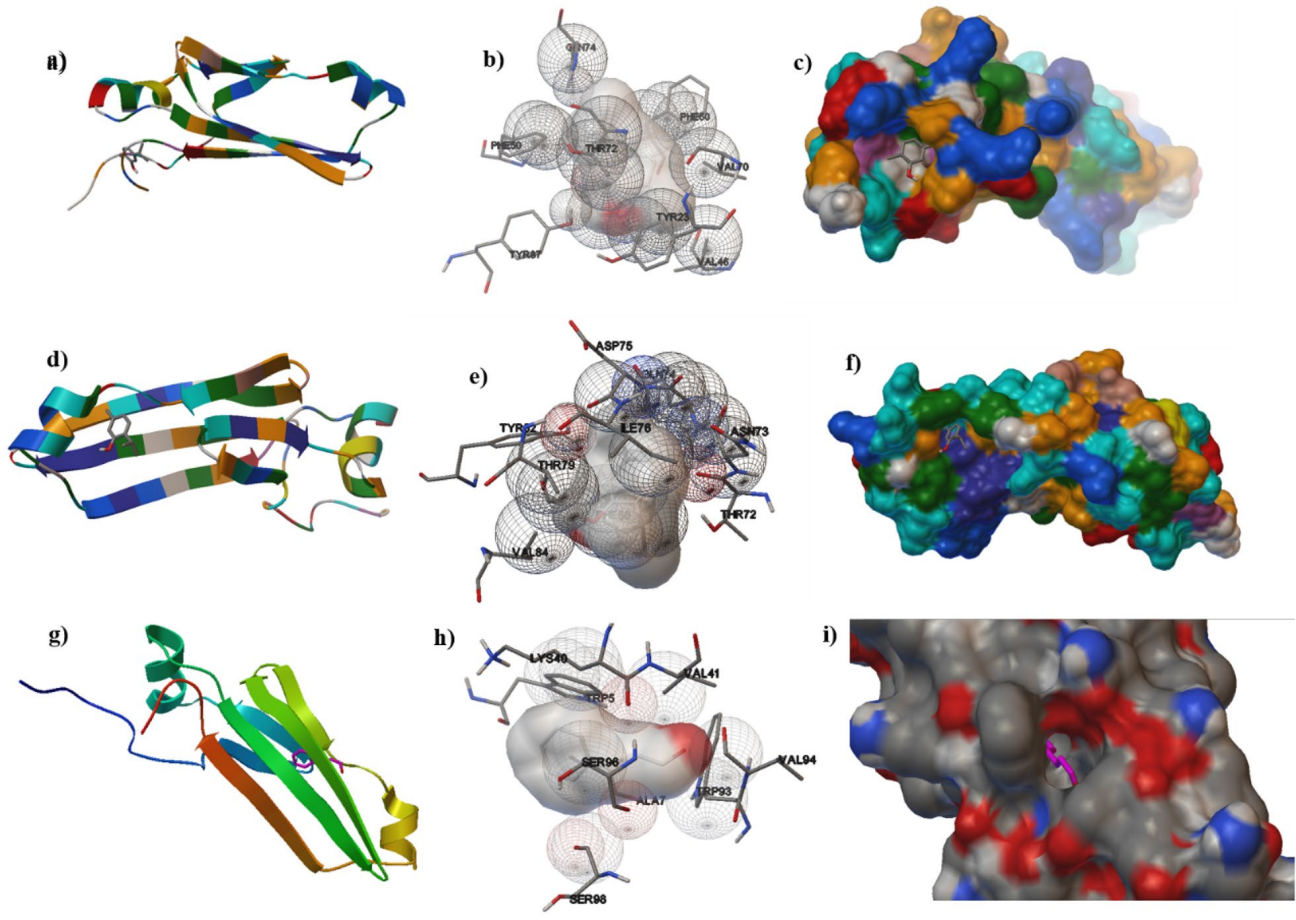
2-D and 3-D interactions of molecular docking of tested phytochemicals inside aap. The horizontal rows denote interactions with carvacrol (**a**–**c**), thymol (**d**–**f**) and cinnamaldehyde (**g**–**i**), respectively; **a**, **d**, **g** denotes secondary structures of protein–protein–ligand complexes; **b**, **e**, **h** protein–ligand interaction, whereas **c**, **f**, **i** denote 3-D conformation of complexes

Protonated low energy 3-D ligand conformation was prepared using Chem 3D v.16.0 software. Further, in silico molecular docking was carried out to estimate the binding energy and to demonstrate the protein–ligand interaction mechanism. All favourably docked structures gained from the molecular docking analysis of the tested phytochemical agents (thymol, carvacrol and cinnamaldehyde) inside the ompC and aap motifs are displayed in Figs. 1 and 2, respectively.

The binding free energy values observed for carvacrol (− 5.27 kcal/mol), cinnamaldehyde (− 5.65 kcal/mol) and thymol (− 4.97 kcal/mol) were nearly equal for both the pathogens (Table 3). Carvacrol was found to interact with OmpC at active sites Leu332, Asp304, Asp33, Asn335 and Thr338, while interacted with aap at Phe60, Val70, Val46, Tyr23, Thr72, Phe50, Tyr87. Moreover, thymol was found to interact with the OmpC at sites Val218, Ala216, Thr239, Phe120 and Asn119, while with aap at Asp75, Ile76, Asn73, Thr72, Thr79 and Val84. Further, cinnamaldehyde interacted with OmpC at the active sites Ser187, Ile182, Phe186 and Tyr226, while with aap at sites Lys40, Val41, Trp5, Ser96, Ala7, Trp93, Ser98 and Val94 (Figs. 1 and 2). In brief, all the three studied phytochemical agents interacted firmly with the respective protein motifs and probably have significance as inhibitors of both aap (EAEC) and ompC (NTS).

**Table 3:** Binding affinity values of tested phytochemicals to dispersin and osmoporin (OmpC) proteins of EAEC and NTS

Sl. No.	Phytochemicals	EAEC			NTS		
		Binding energy (kcal/mol)	Ligand efficiency	Inhibition constant (µM)	Binding energy (kcal/mol)	Ligand efficiency	Inhibition constant (µM)
1.	Carvacrol	− 5.27	− 0.48	137.22	− 4.49	− 0.41	510.49
2.	Cinnamaldehyde	− 5.65	− 0.57	71.61	− 4.65	− 0.47	390.26
3.	Thymol	− 4.97	− 0.45	227.5	− 4.97	− 0.45	226.89

### In vitro antimicrobial efficacy

To corroborate the in silico prediction, MIC and MBC values of the phytochemicals were determined to assess their in vitro antimicrobial efficacy (Table 4). The MIC values of all the three phytochemicals tested ranged from 0.25 to 0.50 µl/ml for MDR–EAEC strains, 0.12 to 0.50 µl/ml for MDR–*S*. Enteritidis strains and for MDR–*S*. Typhimurium strains, it ranged from 0.06 to 0.25 µl/ml. However, the MBC values obtained were either equal to or greater than the MIC values and varied with the strains under study (Table 4).

**Table 4:** MIC and MBC values of phytochemicals against MDR-strains of EAEC and NTS

Isolate ID	Source of isolate	Carvacrol		Cinnamaldehyde		Thymol	
		MIC (µl/ml)	MBC (µl/ml)	MIC (µl/ml)	MBC (µl/ml)	MIC (µl/ml)	MBC (µl/ml)
EAEC (E1)	Infant diarrhoea	0.25	1.0	0.50	0.50	0.25	0.25
EAEC (E2)		0.50	0.50	0.50	1.0	0.25	0.50
EAEC (E3)		0.25	0.50	0.25	0.50	0.25	0.25
*S.* Enteritidis (S1)	Poultry droppings	0.12	0.50	0.12	0.50	0.12	0.50
*S.* Enteritidis (S2)		0.25	0.50	0.12	0.50	0.25	0.50
*S.* Enteritidis (S3)		0.25	0.50	0.12	0.50	0.25	0.50
*S.* Typhimurium (ST1)		0.12	0.50	0.25	0.50	0.25	0.50
*S.* Typhimurium (ST2)		0.12	0.50	0.25	0.50	0.25	0.50
*S.* Typhimurium (ST3)		0.12	0.50	0.12	1.0	0.06	0.50

### In vitro safety assay

A concentration-dependent haemolysis was observed with all the three phytochemicals, both in sheep as well as human ‘O’ erythrocytes. At 1X MIC level, minimal haemolysis was observed both in sheep and human RBCs. However, at 2X and 4X MIC levels, the haemolysis observed was minimal to moderate (less than 40 %), except in carvacrol (57 to 82 %) (Additional file 1: Table S2).

### In vitro stability assay

#### Effect of cationic salts

The MIC values of thymol and cinnamaldehyde remained almost similar for the MDR-EAEC strains even after co-incubating with physiological concentration of cationic salts (150 mM NaCl and 2 mM MgCl_2_). However, a two- to four-fold increase in the MIC levels was observed when MDR-EAEC strains were co-incubated with carvacrol. The MDR–NTS strains tested exhibited a two- to four-fold increase in the MIC values of all the three tested phytochemicals while co-incubating with cationic salts (Additional file 1: Table S3a).

#### Effect of pH

As pH 2.0 did not favour the growth of MDR-EAEC and NTS strains tested, the antimicrobial efficacy for the phytochemicals could not be determined. The MIC values of thymol and cinnamaldehyde remained unaltered with MDR-EAEC strains while treated at varied pH (4.0 to 8.0); however, a two- to four-fold increase in the MIC values was observed with the carvacrol treatment. Nevertheless, the MDR-strains of NTS exhibited a two- to four-fold increase in the MIC values, irrespective of the phytochemicals tested (Additional file 1: Table S3b).

The results of in vitro antimicrobial assay (determination of MIC and MBC values; Table 4) correlated well with the in-silico results (drug likeness, violation to Lipinski’s rule of five) obtained from Swiss ADME data (Table 1). Besides, the results of in silico toxicity assay demonstrated by Protox-II and StopTox softwares (Table 2) were found to be in consonance with the in vitro haemolytic assay results (Additional file 1: Table S2). In short, it was observed that the in silico ADMET analysis correlated well with the in vitro assays performed.

## Discussion

Considering the pace at which drug resistance is emerging among the pathogens of public health significance, drug discovery, design and their development is the need of the hour [22]. Researchers across the globe have been searching for the novel alternative therapeutic strategies or even drug re-purposing [23, 24]. Phytochemicals constitute one such promising alternative [25]. Phytochemicals have been reported to possess a broad-spectrum of antibacterial activities against various pathogens of public health importance [26]. However, in vitro screening of phytochemical compounds for their antibacterial efficacy and toxicity studies would consume more time. Hence, in silico computational approaches associated with chemoinformatics, molecular docking, as well as artificial intelligence, have considerably increased during the past decade in the domain of drug design, development, and discovery [27, 28]. Employing in silico approaches would, therefore, enable virtual screening of molecules which would result in providing better chances of discovering suitable drug candidates in considerably less time and cost. Several structure-based and ligand-based molecular docking approaches are currently available to facilitate high-throughput drug discovery [16, 27]. In the present study, an in silico molecular docking of three identified phytochemicals (thymol, carvacrol, and cinnamaldehyde) to the ompC and aap protein domains of NTS and EAEC strains, respectively was performed. The in-silico data obtained by docking tools was further validated with in vitro antimicrobial efficacy of these phytochemicals against the MDR-strains of NTS and EAEC.

Obnoxious pharmacokinetic properties and toxicity remain hurdles in the development of drug candidates at clinical trials. Hence, identification of suitable candidates with drug-likeness along with sufficient information regarding absorption, distribution, metabolism, excretion, and toxicity (ADMET) is required during the initial phase of drug discovery [28]. The identified phytochemical compounds-monoterpenoids (thymol and carvacrol) and phenylpropanoid (cinnamaldehyde) are generally considered safe for human consumption and have been approved by Food and Drug Administration (FDA) for being used as food additives [18, 19, 21]. However, their ADME profile in connection with in vitro antimicrobial efficacy studies against MDR pathogens has rarely been undertaken. In the present study, the Swiss ADME server was used to analyse various ADME descriptors like, physiochemical properties, pharmacokinetics, solubility, lipophilicity, drug-likeness based on violation of Lipinski’s rule of five and medicinal chemistry [29]. In silico ADME prediction of the three identified phytochemical compounds established drug-likeness as evidenced by no violation to its Lipinski’s rule of five and the obtained bioavailability score (0.55). Further, the red line of the compound in the bioavailability radar plot must be within the pink area to deem the compound as drug-like. The radar plots of thymol and carvacrol were observed completely within the pink area, while that of cinnamaldehyde was fairly within the pink area justifying its drug-likeness. Besides, the ADME data along with the boiled egg model revealed a better gastrointestinal absorption and permeation of the blood-brain barrier by all the tested phytochemicals [29].

It is well understood that the P-gp and cytochrome P450 (CYP) help in biotransformation of xenobiotics to protect tissues [30]. In this study, the phytochemicals exhibited no-P-gp substrate. The P-gp is indicated as the most important member of ATP-binding cassette transporters which is pivotal to evaluate the protective efflux of biological membranes (GI tract or brain) from xenobiotics [29, 31]. Moreover, all the three phytochemicals tested interacted only with CYP1A2 isoenzyme of CYP family, conferring its effectiveness with minimal toxicity [31].

Further, to investigate the in-silico toxicity parameters, Protox-II [32] and StopTox [33] machine learning apps were used. The predicted LD_50_ (mg/kg) for the three phytochemicals tested ranged from 640 to 1850; hence were categorized as toxicity class-4 by Protox-II, indicating that the phytochemicals might be harmful if swallowed (300 < LD_50_ ≤ 2000). Further, all the tested phytocompounds were found to be non-cardiotoxic by StopTox software, based on hERG liability prediction, with the confidence levels ranging between 50 and 60 %. However, the obtained toxicity findings need to be correlated with the in vitro safety assays before their assessment in suitable laboratory animal models.

The in silico ADMET analysis encouraged the phytochemicals to be employed further for Autodock-based computational docking studies [34] to ompC of NTS [35] and aap of EAEC [36]. In this study, ligand-based interaction with the protein domains of pathogens was investigated using blind docking employing Autodock software. Osmoporin (ompC), present in the *Salmonella* spp., is responsible for its survival and pathogenicity and plays a crucial role in diffusing hydrophilic compounds [35]. Moreover, dispersin (aap) present in the EAEC strains is highly immunogenic and represents a class of aggregative factors which are responsible for its functional attribute [36]. The binding free energy values observed for carvacrol, cinnamaldehyde, and thymol were nearly equal for the protein domains of both the pathogens studied. All the studied phytochemical agents interacted firmly with the respective protein motifs and probably have significance as inhibitors of both ompC (NTS) and aap (EAEC). This observed high binding energies obtained during molecular docking would be due to the strong hydrophobic interactions between the phytochemical ligands and the protein motifs-aap and ompC. The in-silico computational data hence obtained needs to be validated with the in vitro antimicrobial efficacy studies.

The antimicrobial efficacy of phytochemicals under study was determined by MIC and MBC values. The MIC values of all the three phytochemicals tested ranged from 0.25 to 0.50 µl/ml for MDR-EAEC strains, 0.06 to 0.50 µl/ml for MDR-NTS strains. However, the MBC values obtained were either equal to or greater than the MIC values. These varied MIC and MBC values of the phytochemicals tested could either be due to strain variation, the difference in the bacterial virulence factors, or structural differences in the bacterial membranes. Moreover, the observed in silico docking results were found to correlate well with the in vitro antimicrobial assays [31, 37].

Further, the computational toxicity assays need to be correlated with the in vitro safety assay, before being passed on for in vivo clinical trials. Therefore, a haemolytic assay based on sheep and human ‘O’ RBCs was employed to ensure the safety profile of phytochemicals. A concentration-dependent haemolytic assay was observed with all three phytochemicals. At 1X MIC level, minimal hemolysis was observed both in sheep and human RBCs; however, at 2X and 4X MIC levels, the haemolysis observed was minimal to moderate, except in carvacrol. An improved specificity of phytochemicals against the bacterial cells and reduction in the haemolytic activity could further be accomplished by increasing the net charge and/or employing conjugation with nanoparticles [38]. The nanotechnological interventions are therefore aimed at delivery of drugs (including phytochemicals) with an intention to effect site-directed drug delivery, reduction in the toxicity with non-compromised safety and therapeutic efficacy by minimizing the concentration of the drug with improved bioavailability [39, 40].

Generally, bioactive phytochemical compounds get degraded in the gastrointestinal tract because of their poor stability [41]; hence, the in vitro stability assays (pH, and cationic salts) were employed for the identified phytochemical compounds. In this study, thymol and cinnamaldehyde tested retained their antimicrobial activities even after subjecting them to varied stability conditions for MDR-EAEC strains, whereas slight decrease in the antimicrobial efficacy was observed against MDR-NTS strains tested. Moreover, carvacrol exhibited a slight increase in the MIC values for all the MDR strains tested. The stability of phytochemical compounds could be improved by nanobiotechnological interventions using a suitable delivery system [41] to get it protected from external as well as biological influences.

## Conclusion

The present study envisaged in silico ADMET analysis, molecular docking as well as in vitro antimicrobial efficacy screening of three identified phytochemical ligands (thymol, carvacrol, and cinnamaldehyde) to the dispersin (aap) and outer membrane osmoporin (ompC) domains of EAEC and NTS, respectively. In silico ADMET prediction and molecular docking studies exhibited a good correlation with the in vitro antimicrobial efficacy studies. The identified phytochemical compounds were further observed to be comparatively stable at different conditions (cationic salts, and pH); however, a concentration-dependent increase in the haemolytic assay was observed against sheep as well as human ‘O’ erythrocytes. Hence, we propose to conduct in silico computation approaches (ADMET analysis, molecular docking) as a high throughput antimicrobial screening tool to provide successful insights for exploring the interaction of phytochemical ligands with various pathogens.

## Materials and methods

### Bacterial strains

The characterised MDR field strains of EAEC (E1; E2; E3), *S*. Enteritidis (S1; S2; S3), and *S*. Typhimurium (ST1; ST2; ST3) maintained in the laboratory repository of Meat Safety Laboratory of ICAR-National Research Centre on Meat, Hyderabad re-validated using PCR assays [42, 43] were used to evaluate the in vitro antibacterial efficacy of phytochemicals. *E. coli* ATCC 25922 was used as the quality control strain.

### Phytochemicals

The phytochemical molecules used in this assay viz., thymol, carvacrol, and cinnamaldehyde (Sigma Aldrich, USA) were retrieved from a literature survey [18–21] for ligand preparation against the protein motifs of the selected pathogens.

### In silico assays

#### Selection of protein motifs

The chemical structures of the identified phytochemical ligands retrieved from the PubChem-NCBI database were drawn using Chem 3D v.16.0 software. The protein motifs of EAEC and NTS selected from the protein data bank (PDB) were dispersin (PDB ID: 2jvu) and outer membrane osmoporin (PDB ID: 3uu2), respectively.

#### In silico absorption, distribution, metabolism, excretion, and toxicity (ADMET) analysis

The identified phytochemicals were analysed for their ADMET analysis using Swiss ADME server (http://www.swissadme.ch/index.php). This server evaluates the compounds for their physicochemical properties, lipophilicity, water solubility, pharmacokinetics, drug likeness and medicinal chemistry. Further, the toxicity properties of the identified phytochemicals were analysed using online servers, Protox-II (https://tox-new.charite.de/protox_II/) [32] and StopTox (https://stoptox.mml.unc.edu/) [33]. Protox-II predicted the LD_50_ (mg/kg) of the identified ligands and toxicity class, whereas StopTox identified cardiotoxicity (hERG liability prediction).

#### Molecular docking using Autodock v.4.20

The protonated low energy 3-D phytochemical ligand conformation was prepared using Chem 3D v.16.0 software. A blind docking employing automated docking software Autodock v.4.20 [34] was used to evaluate the binding affinity of ligands to the aap and ompC motifs of EAEC and NTS, respectively. The target proteins were prepared by removal of ligand, water molecule, hetero atoms, and co-crystallised solvents; non-polar hydrogens were merged and Gasteiger charges and polar-charged hydrogen were added. Further, a grid map was generated with a dimension of 60 × 60 × 60 points with a spacing of 0.375 Å and the Lamarckian genetic algorithm was used to analyse the docking probability. The configuration files created for both the proteins under study generated ten best poses for each of the ligands and scored using Autodock function; the ligands were ranked based on the docked energy. The results of molecular docking were observed using Pymol viewer.

### In vitro assays

#### Determination of minimum inhibitory concentration and minimum bactericidal concentration

The antimicrobial efficacy of identified phytochemicals against multi-drug resistant EAEC and NTS strains was determined by estimating the minimum inhibitory concentration (MIC) and minimum bactericidal concentrations (MBC).

The MIC was determined by incubating 50 µl of the individual test cultures (1 × 10^7^ CFU/ml) in cation-adjusted Mueller Hinton (CA-MH) broth medium (50 µl) with decreasing concentrations of phytochemicals in 96-well flat-bottom microtiter plates for 24 h. Subsequent to the incubation period, resazurin dye (0.015 %) was added to all the wells to determine the dye reduction (pink) and thereby the bacterial inhibition. The lowest concentration of phytochemicals without visible growth was designated as MIC, while the MBC of phytochemicals was estimated by plating 10 µl aliquots from each well revealing no visible growth in MH agar plates (HiMedia, Mumbai, India). The lowest concentration of the phytochemicals which revealed 99.9 % killing of the test culture was defined as the MBC value of the corresponding phytochemical.

#### Safety assays

The haemolytic assay of the individual phytochemical was performed by measuring the release of haemoglobin from sheep and human ‘O’ erythrocytes at 540 nm [44]. The percentage of haemolysis was calculated as (A_sample_ – A_PBS_)/ (A_Triton−X_ – A_PBS_) ×100, in which A_sample_ is the absorbance of phytochemical treatment, A_PBS_ is the absorbance of untreated control with PBS, and A_Triton−X_ is the absorbance of lysed cells treated with Triton X-100 measured at 540 nm.

#### Stability assays

To explore the utility of phytochemicals as therapeutic molecules and their stability, individual phytochemicals were exposed to cationic salts, and different pH levels followed by analysing their MIC and MBC values against test strains as described earlier.

In brief, to investigate the stability of phytochemicals at a physiological concentration of cationic salts (150 mM NaCl and 2 mM MgCl_2_), corresponding phytochemicals were co-incubated in the presence of 150 mM NaCl and 2 mM MgCl_2_, separately with each MDR-strains of pathogens in CA-MH broth [42] and then determined their antimicrobial efficacy. Further, the stability of individual phytochemicals at different pH concentrations (2.0, 4.0, 6.0, and 8.0) was tested by co-incubating each phytochemical and the MDR-strains of pathogens overnight at 37 °C in CA-MH under specific pH.

Subsequent to exposure to varied conditions, the antimicrobial activity of the phytochemicals was estimated by determining their MIC and MBC values against all the selected MDR strains of EAEC, *S*. Enteritidis, and *S*. Typhimurium as described earlier.

## Supplementary Information

**Additional file 1: Table S1.** Antibiotic susceptibility pattern of EAEC and NTS strains under study. **Table S2. **In vitro haemolytic assay of phytochemicals against MDR-strains of EAEC and NTS. **Table S3.** In vitro stability assays (a Cationic salts; b pH) of phytochemicals against MDR-strains of EAEC and NTS. **Fig. S1.** Bioavailability radar of phytochemicals under study. A Thymol; B Carvacrol; C Cinnamaldehyde. **Fig. S2.** Boiled egg graph of phytochemicals under study. A Thymol; B Carvacrol; C Cinnamaldehyde.

## Data Availability

All data generated or analysed during this study are included in this published article [and its supplementary information files].
