# Supplementary material for: In silico molecular docking and in vitro antimicrobial efficacy of phytochemicals against multi-drug-resistant enteroaggregative Escherichia coli and non-typhoidal Salmonella spp
Source: Gut Pathog. 2021 Jul 17;13:46. doi: 10.1186/s13099-021-00443-3 (PMC8286599; doi:10.1186/s13099-021-00443-3)
Supplement: Supplementary file 1 — Additional file 1: Table S1. Antibiotic susceptibility pattern of EAEC and NTS strains under study. Table S2. In vitro haemolytic assay of phytochemicals against MDR-strains of EAEC and NTS. Table S3. In vitro stability assays (a Cationic salts; b pH) of phytochemicals against MDR-strains of EAEC and NTS. Fig. S1. Bioavailability radar of phytochemicals under study. A Thymol; B Carvacrol; C Cinnamaldehyde. Fig. S2. Boiled egg graph of phytochemicals under study. A Thymol; B Carvacrol; C Cinnamaldehyde. [file 13099_2021_443_MOESM1_ESM.docx]

**Supplementary Table 1**. Antibiotic susceptibility pattern of EAEC and NTS strains under study

| **Isolate ID** | Antibiotic Susceptibility testing | | | | | | | | | | MIC Values (µM) | | | | | | | | |
| --- | --- | --- | --- | --- | --- | --- | --- | --- | --- | --- | --- | --- | --- | --- | --- | --- | --- | --- | --- |
|  | CIP | CTR | AMP | COT | TE | IMI | SXT | GEN | CL | CO | CIP | CTR | AMP | COT | TE | IMI | GEN | CL | CO |
| **E1** | S | S | R | R | R | S | R | S | S | S | S | S | >240 | >240 | 60 | S | S | S | S |
| **E2** | R | R | R | R | R | S | S | S | S | S | 120 | >240 | >240 | >240 | 2 | S | S | S | S |
| **E3** | R | R | R | R | R | S | R | S | S | S | 60 | >240 | >240 | >240 | 30 | S | S | S | S |

| **Isolate ID** | Antibiotic Susceptibility testing | | | | | | | | | | | | | | | | | |
| --- | --- | --- | --- | --- | --- | --- | --- | --- | --- | --- | --- | --- | --- | --- | --- | --- | --- | --- |
|  | AMP | CPM | CTX | CPD | AMC | CPZ | GM | CX | K | AT | SF | SD | MRP | AZM | NA | TE | C | NIT |
| **S1** | R | S | S | R | R | S | R | S | R | S | S | R | S | S | R | R | R | S |
| **S2** | R | S | S | R | R | S | R | S | S | S | R | R | S | S | R | S | S | R |
| **S3** | R | S | S | R | R | S | R | S | S | S | S | R | S | S | R | S | S | R |

| **Isolate ID** | Antibiotic Susceptibility testing | | | | | | | | | | | | | | | |
| --- | --- | --- | --- | --- | --- | --- | --- | --- | --- | --- | --- | --- | --- | --- | --- | --- |
|  | AMP | CAZ | COT | CX | AMC | IPM | CIP | CPM | TE | GM | C | LE | NA | CTR | CTX | CZ |
| **ST1** | R | S | R | S | R | S | R | S | R | R | R | R | R | S | S | S |
| **ST2** | R | R | R | R | R | S | R | S | R | R | R | R | R | R | R | S |
| **ST3** | R | I | R | I | R | S | R | S | R | R | R | I | I | S | S | S |

E1, E2, E3: EAEC strains; S1, S2, S3: *Salmonella* Enteritidis strains; ST1, ST2, ST3: *Salmonella* Typhimurium strains. C- Chloramphenicol, LE- Levofloxacin, NA- Nalidicic Acid, CTR- Cefotaxime, CPD- Cefpodoxime, CPZ- Cefoperozone, K- Kanamycin, AT- Aztreonam, SF- Sulphafurazole, SD- Sulfadiazine, MRP- Meropenem, AZM- Azithromycin, NIT- Nitrofurantoin, IMI- Imipenem, SXT- Sulfamethoxazole, CL- Chloramphenicol, CO- Colistin sulphate; S- Sensitive, I- Intermediate, R- Resistant.

**Supplementary Table 2**. *In vitro* haemolytic assay of phytochemicals against MDR- strains of EAEC and NTS.

| **Phytochemicals** | **8X MIC** | **4X MIC** | **2X MIC** | **1X MIC** | **0.50X MIC** | **0.25X MIC** |
| --- | --- | --- | --- | --- | --- | --- |
|  |  |  |  |  |  |  |
| **Sheep RBCs** | | | | | | |
| **Thymol** | 47.576497 | 21.55897 | 11.35002 | 1.489424 | 0.22026432 | 0 |
| **Carvacrol** | 86.008453 | 81.80038 | 75.74179 | 1.245593 | 0.77275051 | 0 |
| **Cinnamaldehyde** | 21.982974 | 12.49039 | 11.74314 | 3.610458 | 0.2744177 | 0.10869565 |
| **Human RBCs** | | | | | | |
| **Thymol** | 48.545882 | 39.37497 | 13.49471 | 2.857837 | 1.91375969 | 1.21698286 |
| **Carvacrol** | 92.679733 | 82.05501 | 57.20799 | 11.23852 | 4.87524225 | 2.65814677 |
| **Cinnamaldehyde** | 19.058586 | 12.76365 | 4.999146 | 2.030588 | 0.69646318 | 0.55358995 |

**Supplementary Table 3**. *In vitro* stability assays (a- Cationic salts; b- pH) of phytochemicals against MDR- strains of EAEC and NTS.

| **Phytochemicals** | EAEC | | | *S.* Enteritidis | | | *S.* Typhimurium | | |
| --- | --- | --- | --- | --- | --- | --- | --- | --- | --- |
|  | E1 | E2 | E3 | S1 | S2 | S3 | ST1 | ST2 | ST3 |
| **150 mM NaCl** | | | | | | | | | |
| **Thymol (µl/ml)** | 0.25 | 0.25 | 0.25 | 0.5 | 0.5 | 1.0 | 0.5 | 0.5 | 0.5 |
| **Carvacrol (µl/ml)** | 1.0 | 1.0 | 1.0 | 0.5 | 1.0 | 0.5 | 0.5 | 0.5 | 0.5 |
| **Cinnamaldehyde (µl/ml)** | 0.5 | 0.5 | 0.5 | 0.5 | 0.5 | 0.5 | 0.5 | 0.5 | 1.0 |
| **2 mM MgCl_2_** | | | | | | | | | |
| **Thymol (µl/ml)** | 0.25 | 0.25 | 0.25 | 0.5 | 0.5 | 0.5 | 0.5 | 1.0 | 0.5 |
| **Carvacrol (µl/ml)** | 0.5 | 1.0 | 1.0 | 0.5 | 0.5 | 0.5 | 0.5 | 0.5 | 0.5 |
| **Cinnamaldehyde (µl/ml)** | 0.5 | 0.5 | 0.5 | 0.5 | 0.5 | 0.5 | 0.5 | 0.5 | 1.0 |

| **Phytochemicals** | **pH** | EAEC | | | *S.* Enteritidis | | | *S.* Typhimurium | | |
| --- | --- | --- | --- | --- | --- | --- | --- | --- | --- | --- |
|  |  | E1 | E2 | E3 | S1 | S2 | S3 | ST1 | ST2 | ST3 |
| **Thymol (µl/ml)** | 4 | 0.25 | 0.25 | 0.25 | 0.5 | 0.5 | 0.5 | 0.5 | 0.5 | 0.5 |
|  | 6 | 0.25 | 0.25 | 0.25 | 0.5 | 0.5 | 0.5 | 0.5 | 1.0 | 0.5 |
|  | 8 | 0.5 | 0.25 | 0.5 | 0.5 | 1.0 | 0.5 | 0.5 | 1.0 | 1.0 |
| **Carvacrol (µl/ml)** | 4 | 0.5 | 1.0 | 0.5 | 0.5 | 0.5 | 0.5 | 0.5 | 0.5 | 1.0 |
|  | 6 | 0.5 | 1.0 | 0.5 | 0.5 | 0.5 | 0.5 | 0.5 | 1.0 | 1.0 |
|  | 8 | 0.5 | 1.0 | 0.5 | 0.5 | 0.5 | 0.5 | 2.0 | 1.0 | 1.0 |
| **Cinnamaldehyde (µl/ml)** | 4 | 0.5 | 0.5 | 0.5 | 0.5 | 0.5 | 0.5 | 0.5 | 1.0 | 1.0 |
|  | 6 | 0.5 | 0.5 | 0.5 | 0.5 | 0.5 | 0.5 | 0.5 | 1.0 | 1.0 |
|  | 8 | 0.5 | 0.5 | 0.5 | 0.5 | 0.5 | 1.0 | 0.5 | 1.0 | 1.0 |


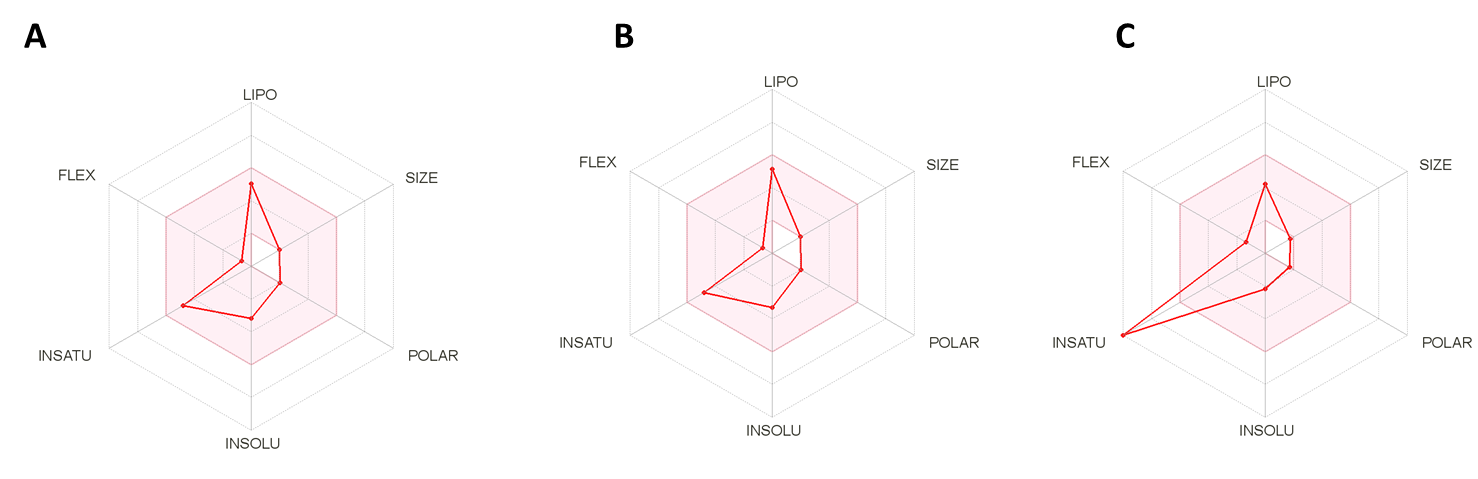


**Supplementary Fig. 1** Bioavailability radar of phytochemicals under study. A- Thymol; B- Carvacrol; C- Cinnamaldehyde


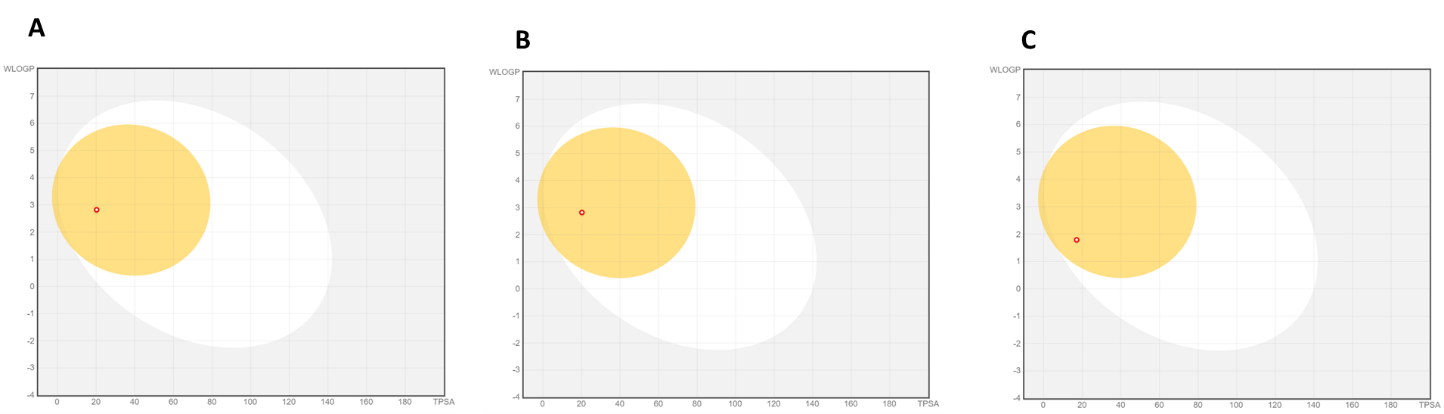


**Supplementary Fig. 2** Boiled egg graph of phytochemicals under study. A- Thymol; B- Carvacrol; C- Cinnamaldehyde
